# The C-terminal regions of the GLP-1 and GIP receptors are not the key determinants of their differential arrestin recruitment but modulate the rate of receptor endocytosis

**DOI:** 10.3389/fphar.2025.1528295

**Published:** 2025-03-25

**Authors:** Bashaier Al-Zaid, Suleiman Al-Sabah

**Affiliations:** Department of Pharmacology and Toxicology, Faculty of Medicine, Kuwait University, Kuwait City, Kuwait

**Keywords:** glucagon-like polypeptide-1, glucose-dependent insulinotropic polypeptide, G protein-coupled receptor, arrestin, endocytosis

## Abstract

**Introduction:** Glucagon-like peptide-1 (GLP-1) and glucose-dependent insulinotropic polypeptide (GIP) are important regulators of metabolism and mediate the incretin effect. This glucose-dependent potentiation of insulin secretion is severely impaired in patients with type-2 diabetes mellitus. While pharmacological doses of GLP-1 can overcome this impairment, the same is not true for GIP. The reasons for this are unclear. However, differences in the signalling profiles of the GLP-1 and GIP receptors (GLP-1R and GIPR) may contribute. GLP-1R and GIPR are closely related G protein-coupled receptors but differ in their ability to recruit arrestin, GIPR being relatively poorer. Furthermore, these receptors have been reported to utilize different mechanisms to undergo agonist-induced internalization.

**Methods:** This study aimed to identify the role of the C-terminal region of the two receptors in their differing signalling behaviour using chimeric receptors where the C-terminal tail of one receptor was replaced with that of the other.

**Results:** Replacement of the C-terminal tail had only limited effects on G protein and arrestin recruitment to either receptor. GIP-stimulated internalisation of GIPR occurred at a significantly (*P* < 0.001) slower rate than GLP-1-stimulated internalisation of GLP-1R. Replacement of the C-terminal tail of GIPR with that of GLP-1R significantly (*P* < 0.05) increased the internalization rate but not to the rate of wild-type GLP-1R. The reciprocal substitution significantly (*P* < 0.005) decreased internalization rate.

**Conclusion:** These data show that the C-terminal region of GLP-1R and GIPR is not the critical determinant of their differing ability to recruit arrestin but modulates receptor endocytosis.

## 1 Introduction

Drugs that activate the glucagon-like peptide-1 (GLP-1) receptor are currently used clinically to treat type 2 diabetes mellitus (T2DM) and obesity (Nauck et al., 2021). In contrast, it is only recently that a drug, which also activates the receptor for the other incretin, glucose-dependent insulinotropic polypeptide (GIP), has been approved for the treatment of T2DM (i.e., tirzepatide, a dual GLP-1/GIP receptor agonist) (Le Roux et al., 2023). The primary action of GLP-1 and GIP is to potentiate insulin secretion in a glucose-dependent manner, a phenomenon referred to as the incretin effect (Mcintyre et al., 1964; Nauck et al., 1986). The incretin effect can account for at least 40% of the insulin produced postprandially in healthy individuals, and a loss of response to both endogenous GLP-1 and GIP, along with the subsequent deterioration of the incretin effect, is an early characteristic of T2DM (Holst et al., 2011; Gasbjerg et al., 2019). Pharmacological doses of GLP-1 can overcome this impairment in subjects with T2DM, but the same is not observed with GIP, even though in healthy individuals, GIP contributes more to the incretin effect than GLP-1 (Nauck et al., 1993; Gasbjerg et al., 2020).

The GLP-1 and GIP receptors (GLP-1R and GIPR, respectively) are closely related members of family B or the secretin class of G protein-coupled receptors (GPCRs) and share a high degree of sequence homology (Mayo et al., 2003; Graaf et al., 2016). Both receptors are expressed in pancreatic β-cells and other cell types, resulting in numerous pleiotropic effects of their peptide agonists. For example, GLP-1 decreases appetite and impairs glucagon secretion, and GIP regulates bone and adipocyte metabolism (Mayendraraj et al., 2022; Liu et al., 2022; Drucker and Holst, 2023). Studies showing that GIPR-knockout mice were resistant to weight gain when fed a high-fat diet suggested that blocking the actions of GIP may be a viable strategy for treating obesity (Miyawaki et al., 2002). This observation led to the development of (Pro3)GIP, an analog of GIP with a glutamic-acid-to-proline substitution at position 3, improving glucose homeostasis and preventing weight gain in animal models of diabetes and obesity (McClean et al., 2007; Gault et al., 2007). Although (Pro3)GIP was initially considered an antagonist, it was subsequently shown to be a low-potency agonist (Al-Sabah et al., 2014a; Sparre-Ulrich et al., 2016). Despite this finding, GIPR antagonists continued to be developed as candidates for treating T2DM and obesity. Maridebart cafraglutide (MariTide), a fully human monoclonal anti-human GIPR antagonist antibody conjugated to two GLP-1R agonist peptides, has recently exhibited robust weight loss in subjects with obesity in a phase 2 study (Véniant et al., 2024; Amgen, 2024). In contrast, tirzepatide, a dual GIPR/GLP-1R agonist, has been approved for the treatment of T2DM and additionally produces significant weight loss (Le Roux et al., 2023). It is currently unclear why both GIPR agonists and antagonists improve glucose control and reduce body weight. It is possible that the agonists act as functional antagonists by desensitizing the receptor, as demonstrated by Killion et al. (2020) and, more recently, by Davies et al. (2025).

The reasons why GLP-1, but not GIP, remains insulinotropic in T2DM are still unclear but may be related to the receptors’ differing signaling profiles (Yabe and Seino, 2011; Al-Sabah, 2016). GLP-1R and GIPR signal primarily through G_αs_, resulting in an increase in intracellular cyclic adenosine monophosphate (cAMP). GLP-1R can also signal through G_αq_, which may enable this receptor to function under conditions of hyperglycemia (Oduori et al., 2020), a hypothesis challenged by the observation that GIPR has also been shown to signal through G_αq_ (Manchanda et al., 2023). The two receptors also appear to differ in their kinetics and mechanisms of internalization and desensitization (Manchanda et al., 2023). GLP-1R undergoes rapid endocytosis following agonist stimulation, whereas GIPR has been reported to constitutively recycle between the cell surface and intracellular compartments (Shaaban et al., 2016; Mohammad et al., 2014). Although GLP-1R interacts with arrestin, its role in receptor endocytosis is still undetermined; nevertheless, knockdown experiments show that arrestin is involved in GLP-1-mediated insulin secretion (Sonoda et al., 2008). In contrast, how well GIPR interacts with arrestin is a subject of continuing investigation, with several conflicting studies in the literature (Al-Sabah et al., 2014b; Ismail et al., 2015; Gabe et al., 2018).

In this study, we investigated the role of the C-terminal tails of GLP-1R and GIPR in their differing signaling properties using chimeric receptors, where the C-terminal region of one receptor was replaced with that of the other. We hypothesized that replacing GIPR’s C-terminal tail with that of GLP-1R would result in a receptor that signals and internalizes like GLP-1R and *vice versa*.

## 2 Materials and methods

### 2.1 Materials

All peptide ligands were purchased from Bachem (Bubendorf, Switzerland). Cell culture reagents were purchased from Gibco-Invitrogen (Paisley, United Kingdom) and Sigma-Aldrich (Poole, United Kingdom). General chemicals were purchased from Sigma-Aldrich.

### 2.2 Construction of cDNA

cDNA encoding the following constructs has been previously described: C-terminally labeled super yellow fluorescent protein 2 (SYFP2) and Nano Luciferase (NLuc)-labeled human GLP-1R and GIPR, arrestin3–NLuc, arrestin3–SYFP2 (Arr3–SYFP2), and mCherry CAAX (Al-Zamel et al., 2019; Al-Sabah et al., 2020). Gibson assembly was employed to generate chimeric receptors, where the C-terminal region of GLP-1R was replaced with that of GIPR and *vice versa* (Table 1). For this procedure, the NEBuilder^®^ HiFi DNA Assembly Cloning Kit was purchased from New England Biolabs (Ipswich, Massachusetts, USA). In brief, the N-terminal and transmembrane region of one receptor and the C-terminal tail of the other receptor were amplified with primers that introduced a HindIII restriction site directly upstream of the start codon of the receptor and replaced the stop codon with an XbaI restriction site (primers are shown in Supplementary Table 1). To produce the vector, GLP-1R-SYFP2 in pcDNA 3.1 was digested with HindIII and XbaI. The resulting PCR products and the vector were purified, and the Gibson assembly reaction was set up on ice to include 100 ng of the vector, 500 ng of the insert, and 10 µL of the NEBuilder HiFi DNA Assembly Master Mix. The volume was adjusted to 20 µL with deionized distilled water. The samples were incubated at 50°C for 15–60 min and then chilled on ice or stored at −20°C. Finally, 2 µL of the chilled assembled products were transformed into competent *E. coli.* Ampicillin was used to select colonies, and the purified plasmids resulted in GLP 1/GIPR-SYFP2 and GIP/GLP-1R-SYFP2. To label these chimeric receptors at their C-termini with either NLuc or RLuc8, the plasmid was digested with HindIII and XbaI, and the purified fragment was ligated into either Arr3-NLuc or the GLP-1 receptor labeled at the C-terminus with RLuc8 (GLP-1R–RLuc8) that had previously been digested with HindIII and XbaI, replacing the Arr3 or GLP-1R open reading frame with that of the chimeric receptor. Wild-type GIPRs were labeled at their C-termini with RLuc8 using the same strategy.

**TABLE 1:** Recruitment of Venus-labeled mG_αs_ or mG_αq_ subunits to NLuc-labeled receptors.

		Venus-mG_s_	Venus-mG_s_	Venus-mG_q_	Venus-mG_q_
Receptor	*Agonist*	*p*EC_50_	*E*_*MAX*_	*p*EC_50_	E_MAX_
GLP-1R	*GLP-1*	7.6 ± 0.06 _(3)_	1.15 ± 0.01 _(3)_	7.3 ± 0.13 _(3)_	1.08 ± 0.02 _(3)_
GLP-1/GIPR	*GLP-1*	7.5 ± 0.07 _(3)_	1.21 ± 0.01 _(3)_	7.1 ± 0.13 _(3)_	1.07 ± 0.02 _(3)_
GIPR	*GIP*	8.9 ± 0.20 _(4)_	1.07 ± 0.02 _(4)_ ^a^ ^,^ ^c^	8.6 ± 0.16 _(5)_	1.03 ± 0.003 _(5)_ ^b^
GIP/GLP-1R	*GIP*	8.9 ± 0.41 _(3)_	1.06 ± 0.001 _(3)_ ^a^ ^,^ ^c^	9.0 ± 0.19 _(3)_	1.04 ± 0.001 _(3)_ ^a^ ^,^ ^b^

The data represent the mean ± SEM, from at least three independent experiments (the number of experiments is shown in parentheses) performed in triplicate. *p*EC_50_ refers to −log EC_50_/M. The *E*_*MAX*_ value indicates the maximum BRET signal as fold-change from baseline.

^a^ *P* < 0.05 significantly different from wild-type GLP-1R.

^b^ *P* < 0.01.

^c^ *P* < 0.005 significantly different from GLP-1/GIPR. The *E*_*MAX*_ value indicates the maximum BRET signal expressed as fold change from baseline.

GLP-1R–RLuc8 (Jorgensen et al., 2011) was a kind gift from Rasmus Jorgensen (Novo Nordisk, Denmark). NES-Venus-mG_αs_, NES-Venus-mG_αq_, and Venus-KRAS (Wan et al., 2018) were kind gifts from Mohammed Ayoub (Khalifa University, Abu Dhabi, United Arab Emirates).

All constructs were verified through Sanger sequencing.

### 2.3 Cell culture and transfection of cells

HEK-293 cells were cultured in Dulbecco’s modified Eagle’s media, supplemented with 10% fetal calf serum, 100 U/mL penicillin, and 100 μg/mL streptomycin. Cells were maintained at 37°C in a humidified environment containing 5% CO_2_. HEK-293 cells were transiently transfected using Effectene (QIAGEN, Hilden, Germany), following the manufacturer’s protocol.

### 2.4 Bioluminescence resonance energy transfer assays

To generate dose-response curves, HEK-293 cells were transiently transfected with equal amounts of labeled receptors and labeled G protein for G protein recruitment assay and in a 1:2 ratio for arrestin and KRAS assays. HEK 293 cells were transiently co-transfected with either NES-Venus-G_αs_, NES-Venus-G_αq_, or NLuc/SYFP2-labeled Arr3 and a labeled (NLuc, RLuc8, or SYFP2) receptor. The total amount of DNA did not exceed 2 µg in a 1:1 (receptor: G protein) or 1:2 (receptor: Arr3 or KRAS) ratio. Forty-eight hours post-transfection, cells were detached and washed with Hank’s Balanced Salt Solution (HBSS). Cells were re-suspended in HBSS and plated onto white 96-well plates (PerkinElmer) in suspension at a density of 180,000 cells/well. Cells were incubated with the agonist for 15 min (except in the case of KRAS assays, where the incubation time was 60 min), and bioluminescence resonance energy transfer (BRET) measurements were taken using a Victor X4 plate reader (PerkinElmer) immediately after the addition of coelenterazine h (final concentration: 1 μM). NLuc/RLuc8 emission was measured using a 460/40-nm filter, and the resulting SYFP2 emission was read using a 535/25-nm filter. To generate dose-response curves, the BRET ratio was expressed as fold-change from non-stimulated, and curves were fitted using a “sigmoidal dose-response curve” function.

In BRET kinetic studies, HEK-293 cells were transiently transfected with cDNA encoding for RLuc8-labeled receptor and Venus-labeled KRAS in a 1:2 ratio. Forty-eight hours post-transfection, cells were detached and washed with and re-suspended in HBSS. Cell suspensions were plated onto white 96-well plates (PerkinElmer) at a density of 180,000 cells/well, and coelenterazine *h* was added (final concentration: 1 μM). Basal BRET was measured for 10–20 min before adding the agonist or vehicle, after which BRET was continuously measured for an additional 60 min. Kinetic BRET measurements were taken at 37°C using a Spark plate reader (Tecan) with 460/40 nm and 535/25 nm filters for RLuc8 and Venus emissions, respectively. The BRET signal resulting from ligand stimulation was calculated by subtracting the BRET ratio for the vehicle-treated cells from the ligand-stimulated cells of the same aliquot. This calculation is needed as the vehicle-treated cells represent the background. The final pretreatment reading is presented at the zero-time point.

### 2.5 Confocal microscopy

HEK-293 cells transiently expressing SYFP2-labeled receptors and mCherry-CAAX (at a ratio of 3:2) or RLuc8-labeled receptors and Arr3-SYFP2 (at a ratio of 3:1) were plated onto a poly-D-lysine-coated coverslip and mounted onto an “Attofluor” holder (Molecular Probes, Leiden, Netherlands). The cellular location of the labeled receptors was monitored by live cell confocal microscopy performed using a Zeiss LSM 900 Meta System (Carl Zeiss, Oberkochen, Germany). Zeiss Zen Blue 2 software (2.1) was used for data acquisition and analysis. Images were taken with an oil-immersion 63× lens using the factory settings for mCherry and YFP.

### 2.6 Data analyses

Dose-response data were fitted to a sigmoidal curve using GraphPad 9.0 (GraphPad, San Diego, CA). The values are expressed as the mean ± standard error of the mean; n represents the number of independent experiments. Statistical significance was calculated using an unpaired, two-tailed Student’s t-test or ANOVA, followed by Tukey’s *post hoc* test where appropriate, using GraphPad 9.0.

## 3 Results

### 3.1 G protein recruitment to wild-type and chimeric receptors

**FIGURE 1:**
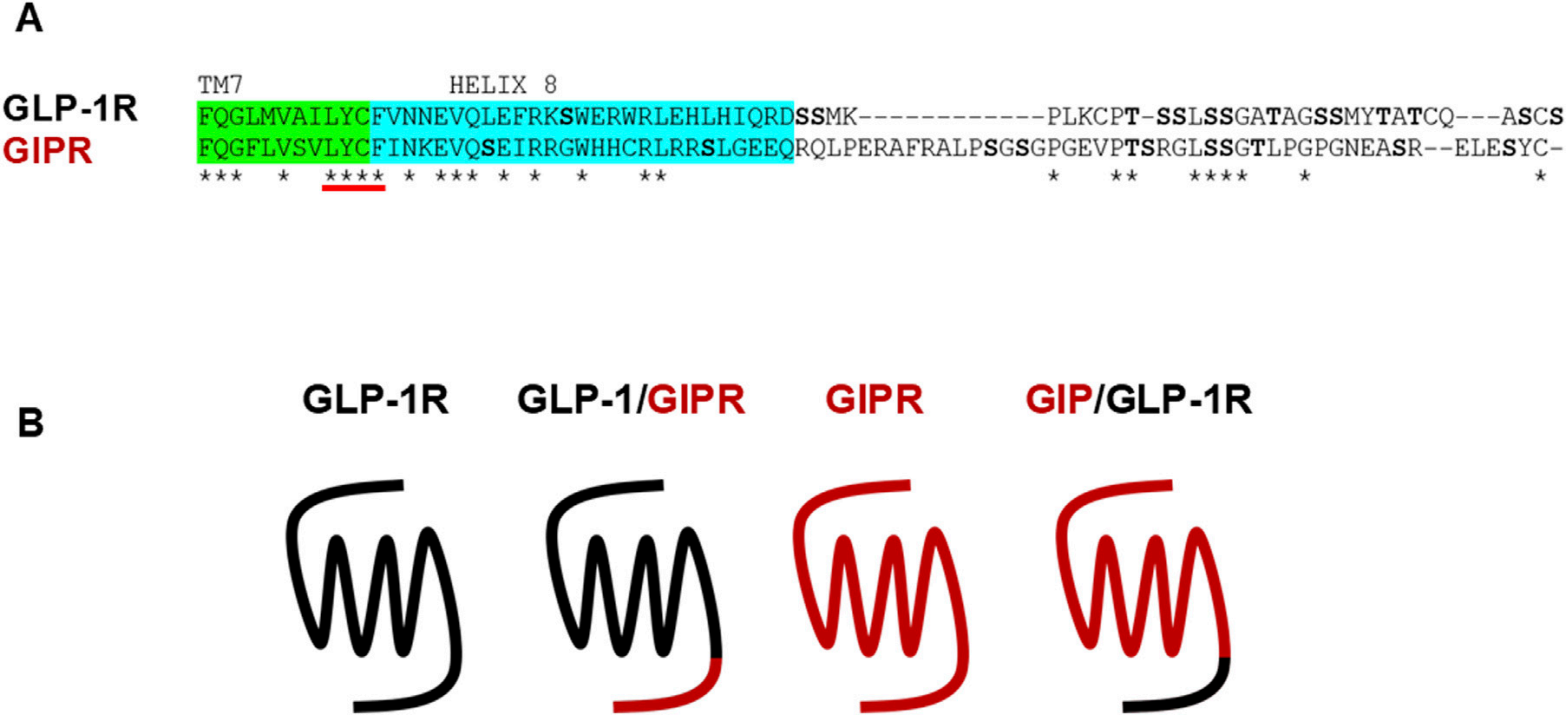
Wild-type and chimeric receptors used in this study. **(A)** Sequence alignment of the C-terminal regions of GLP-1R and GIPR. Potential phosphorylation sites are shown in bold. Sequence immediately following transmembrane helix 7 was substituted to generate the chimeric receptors. **(B)** Schematic representation of the wild-type and chimeric receptors used in this study.

**FIGURE 2:**
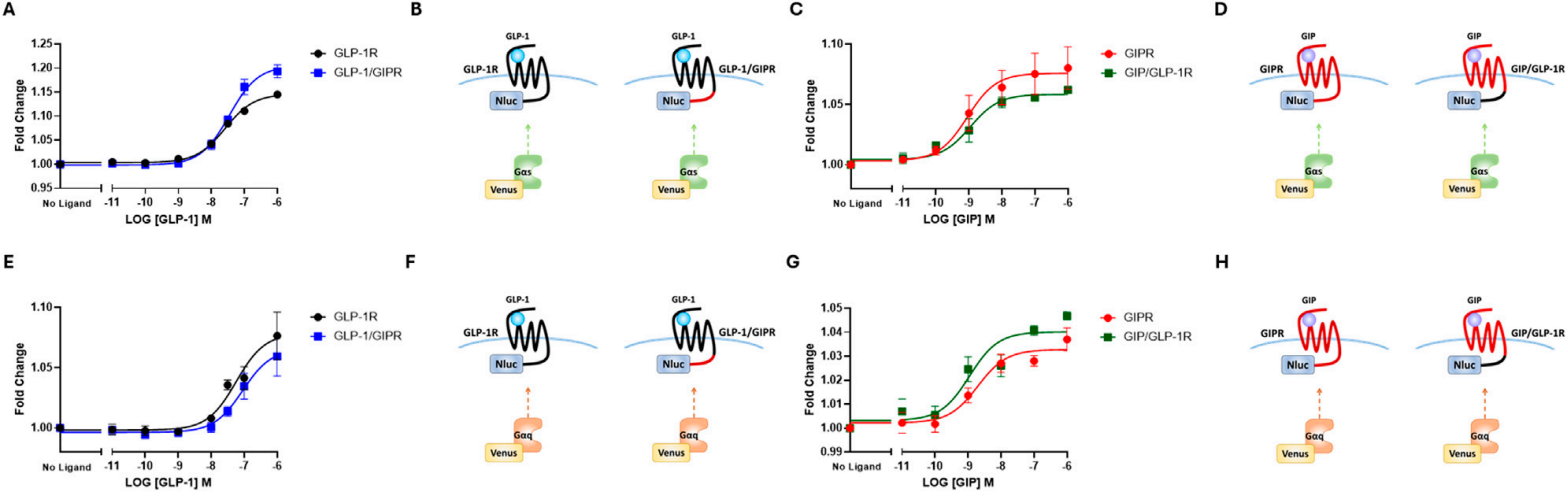
The replacement of C-terminal region does not impact G-protein recruitment. **(A)** Concentration-dependent Venus-mG_αs_ recruitment to NLuc-labeled wild-type or chimeric GLP-1R stimulated by GLP-1. **(B)** Schematic representation of the chimeric receptors, BRET pairs, and ligands used in **(A)**. **(C)** Concentration-dependent Venus-mG_αs_ recruitment to NLuc-labeled wild-type or chimeric GIPR stimulated by GIP. **(D)** Schematic representation of the chimeric receptors, BRET pairs, and ligands used in **(C)**. **(E)** Concentration-dependent Venus-mG_αq_ recruitment to NLuc-labeled wild-type or chimeric GLP-1R stimulated by GLP-1. **(F)** Schematic representation of the chimeric receptors, BRET pairs, and ligands used in **(E)**. **(G)** Concentration-dependent Venus-mG_αq_ recruitment to NLuc-labeled wild-type or chimeric GIPR stimulated by GIP. **(H)** Schematic representation of the chimeric receptors, BRET pairs, and ligands used in **(G)** Data represent the mean ± S.E.M displayed as error bars, from at least three independent experiments, each performed in triplicate.

To investigate the impact of substituting the C-terminal region of one receptor with that of the other, HEK-293 cells were transiently transfected with either the wild-type or chimeric receptor (shown schematically in Figure 1B) labeled with NLuc and Venus-labeled mini-G proteins. BRET assays were used to generate dose-response curves. GLP-1 stimulated Venus-mG_αs_ to GLP-1R in a dose-dependent manner. Substituting the C-terminal region of GLP-1R with that of GIPR had no significant effect on either E_*MAX*_ or potency (Figure 2A; Table 1). The reciprocal substitution had no significant effect on GIP’s ability to recruit Venus-mG_αs_ to GIPR (Figure 2C; Table 1). Similar results were found when Venus-mG_αq_ recruitment was investigated. Substituting GLP-1R’s C-tail with that of GIPR did not significantly affect either E_*MAX*_ or *p*EC_50_ (Figure 2E; Table 1) and vice versa (Figure 2G; Table 1).

### 3.2 Arrestin recruitment to wild-type and chimeric receptors

**FIGURE 3:**
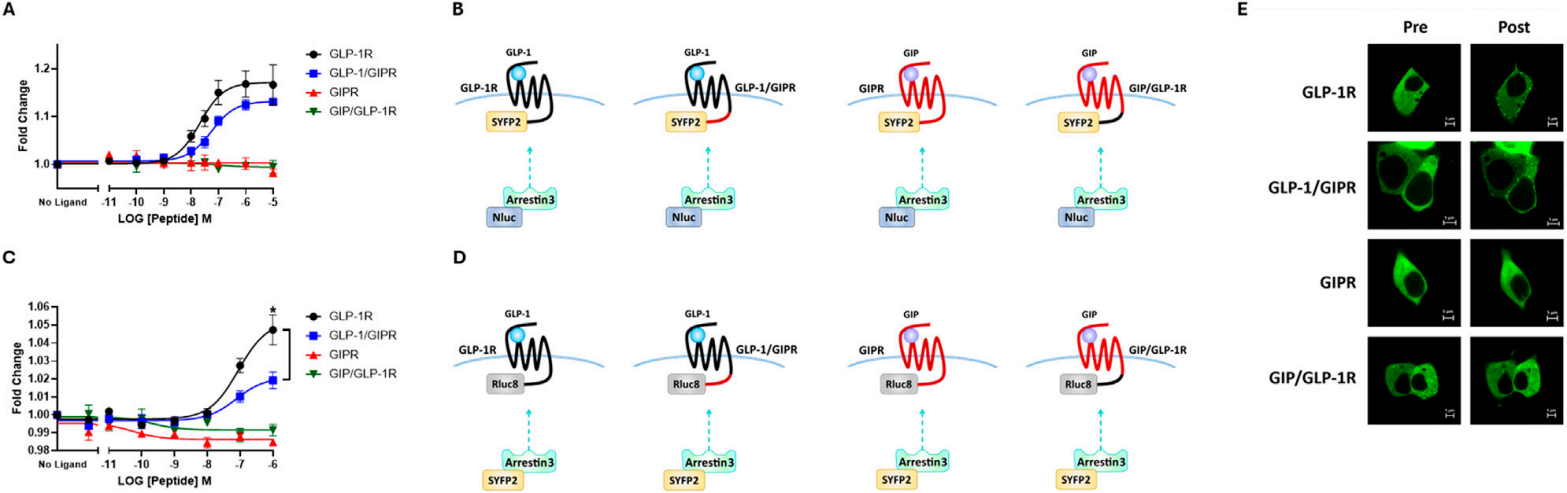
The replacement of the C-terminal region of GIPR with that of GLP-1R does not improve arrestin recruitment to GIPR. **(A)** Concentration-dependent Arr3–NLuc recruitment to SYFP2-labeled wild-type or chimeric receptors stimulated by GLP-1 or GIP. **(B)** Schematic representation of the chimeric receptors, BRET pairs, and ligands used in **(A)**. **(C)** Concentration-dependent Arr3–SYFP2 recruitment to RLuc8-labeled wild-type or chimeric receptors stimulated by GLP-1 or GIP. **(D)** Schematic representation of the chimeric receptors, BRET pairs, and ligands used in **(C)**. Data represent the mean ± S.E.M displayed as error bars, from at least three independent experiments, each performed in triplicate. *P < 0.05. **(E)** HEK-293 cells were transiently transfected with Arr3–SYFP2 and NLuc-labeled receptors. Confocal images were captured immediately before and 15 min after treatment with 1 μM GLP-1 (for GLP-1R and GLP-1/GIPR) or GIP (for GIPR and GIP/GLP-1R). The images are representative of at least three independent experiments. Scale bar: 5 μm.

NLuc-labeled arrestin3 recruitment to SYFP2-labeled GLP-1R and GIPR was investigated using BRET. Substituting GLP-1R’s C-terminal tail with that of GIPR had no significant effect on either the potency or the extent to which GLP-1 stimulated arrestin3 recruitment to GLP-1R (Figure 3A; Table 2). Interestingly, when using a different BRET pair (RLuc8-labeled receptor and SYFP2-labeled arrestin3), a small but significant (*P* < 0.05) decrease in *E*_*MAX*_ was observed, but no change was observed in potency (Figure 3C; Table 2). Stimulating GIPR with GIP did not result in any detectable arrestin3 recruitment in this assay, even at concentrations of 10 µM. Substituting the C-terminal region of GIPR with that of GLP-1 had no detectable impact on GIP-stimulated arrestin3 recruitment to GIPR using either BRET pair (Figures 3A, C; Table 2).

**TABLE 2:** Recruitment of NLuc-labeled arrestin3 to SYFP2-labeled receptors and SYFP-2-labeled arrestin3 to RLuc8-labeled receptors.

		Arr3-NLuc	Arr3-NLuc	Arr3-SYFP2	Arr3-SYFP2
Receptor	*Agonist*	*p*EC_50_	*E*_*MAX*_	*E*_*MAX*_	pEC_50_
GLP-1R	*GLP-1*	7.6 ± 0.11 _(8)_	1.18 ± 0.03 _(8)_	1.05 ± 0.09 _(5)_	7.07 ± 0.02 _(5)_
GLP-1/GIPR	*GLP-1*	7.3 ± 0.14 _(5)_	1.13 ± 0.01 _(5)_	1.02 ± 0.01 _(4)_ ^a^	7.18 ± 0.10 _(4)_
GIPR	*GIP*	ND	ND	ND	ND
GIP/GLP-1R	*GIP*	ND	ND	ND	ND

The mean ± SEM is shown from at least three independent experiments (the number of experiments is shown in parentheses) performed in triplicates. ND refers to assays that exhibited no detectable activity. *p*EC_50_ refers to −log EC_50_/M. The *E*_*MAX*_ value indicates the maximum BRET signal expressed as fold-change from baseline.

^a^ *P* < 0.05 significantly different from wild-type GLP-1R.

These results were supported by confocal microscopy experiments. HEK-293 cells were transiently transfected with SYFP2-labeled arrestin3 and NLuc-labeled receptor. Images were captured immediately before and 15 min after stimulation with 1 µM GLP-1, for GLP-1R and GLP-1R/GIPR, or 1 µM GIP, for GIPR and GIP/GLP-1R. Prior to agonist stimulation, Arr3-SYFP2 was visible in the cytosol, regardless of the receptor it was co-transfected with. Following stimulation with GLP-1, Arr3-SYFP2 translocation to the plasma membrane was clearly visible in cells expressing either wild-type GLP-1R or GLP-1/GIPR. No observable arrestin translocation following agonist stimulation was detectable in cells expressing either GIPR or GIP/GLP-1R (Figure 3E).

### 3.3 Cell surface expression of wild-type and chimeric receptors

**FIGURE 4:**
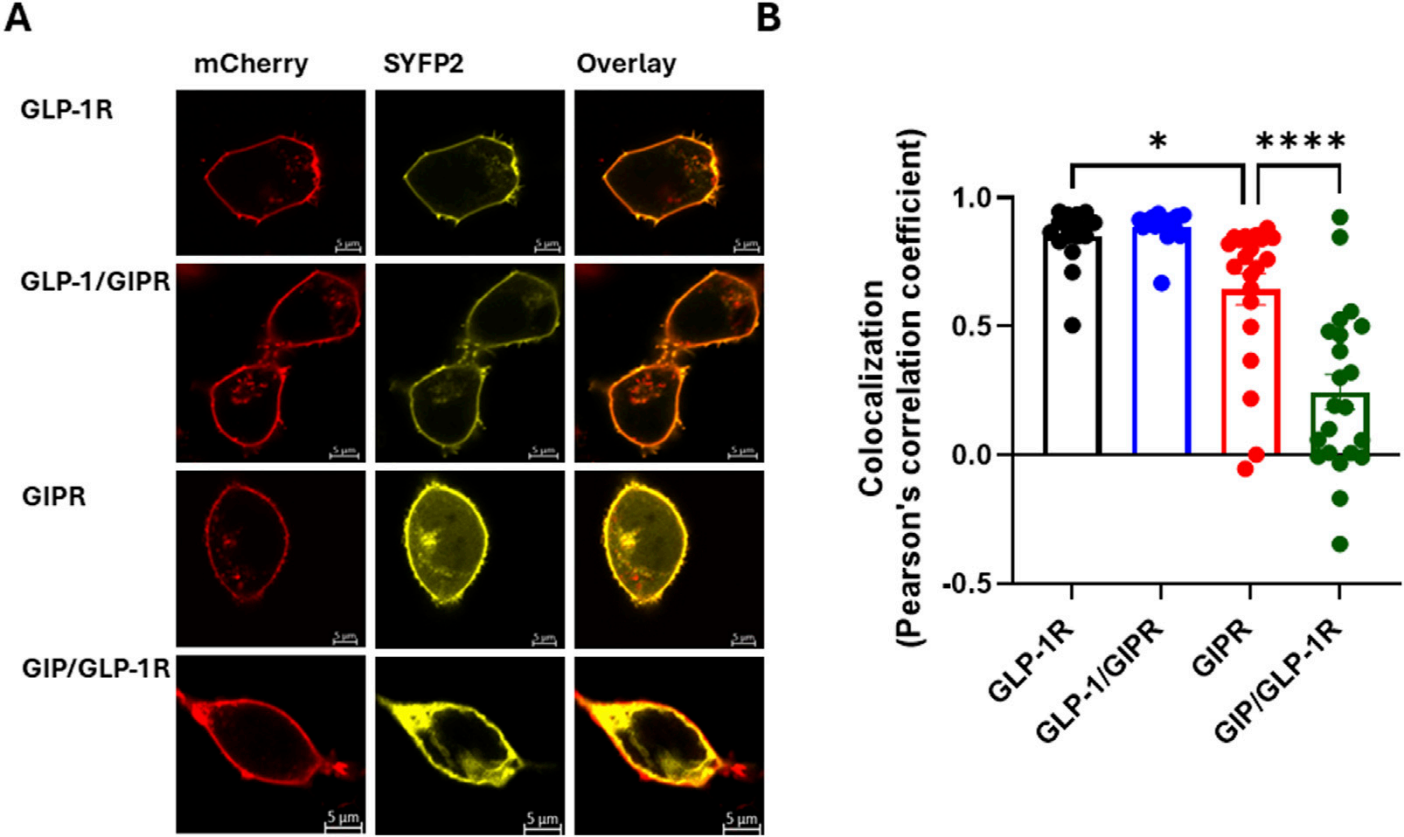
Cell surface expression of wild-type and chimeric GLP-1R and GIPR **(A)**. Representative live cell images of HEK-293 cells transiently co-transfected with plasma membrane-targeted mCherry-CAAX (red) and SYFP2-labeled receptor (yellow) visualized by confocal microscopy. GLP-1R-SYFP2 and GLP-1/GIPR-SYFP2 appear to be expressed primarily at the plasma membrane, while GIPR-SYFP2 and GIP/GLP-1R-SYFP2 are found not only at the plasma membrane but also in the cytosol. **(B)** The exchange of the C-terminal region neither affected the surface expression of GLP-1/GIPR nor enhanced the surface expression of GIP/GLP-1R compared to the corresponding wild-type receptor. The mean ± S.E.M displayed as error bars, from at least three independent experiments, and the images are representative of at least three independent experiments. Scale bar: 5 μm.

The impact of substituting the C-terminal region of one receptor with that of the other on cell surface expression was assessed using confocal microscopy and the colocalization of SYFP2-labeled receptor with a membrane-targeted red fluorescent protein (mCherry-CAAX). GLP-1R colocalized with mCherry-CAAX to a high degree, and substituting this receptor’s C-terminal region with that of GIPR had no significant impact on this colocalization. GIPR, on the other hand, colocalized significantly less well with mCherry-CAAX than either wild-type GLP-1R or GLP-1/GIPR (*P* < 0.05). Substituting GIPR’s C-tail with that of GLP-1R significantly (*P* < 0.001) decreased colocalization compared to all other receptors investigated (Figures 4A, B).

### 3.4 Agonist-stimulated endocytosis of wild-type and chimeric receptors

**FIGURE 5:**
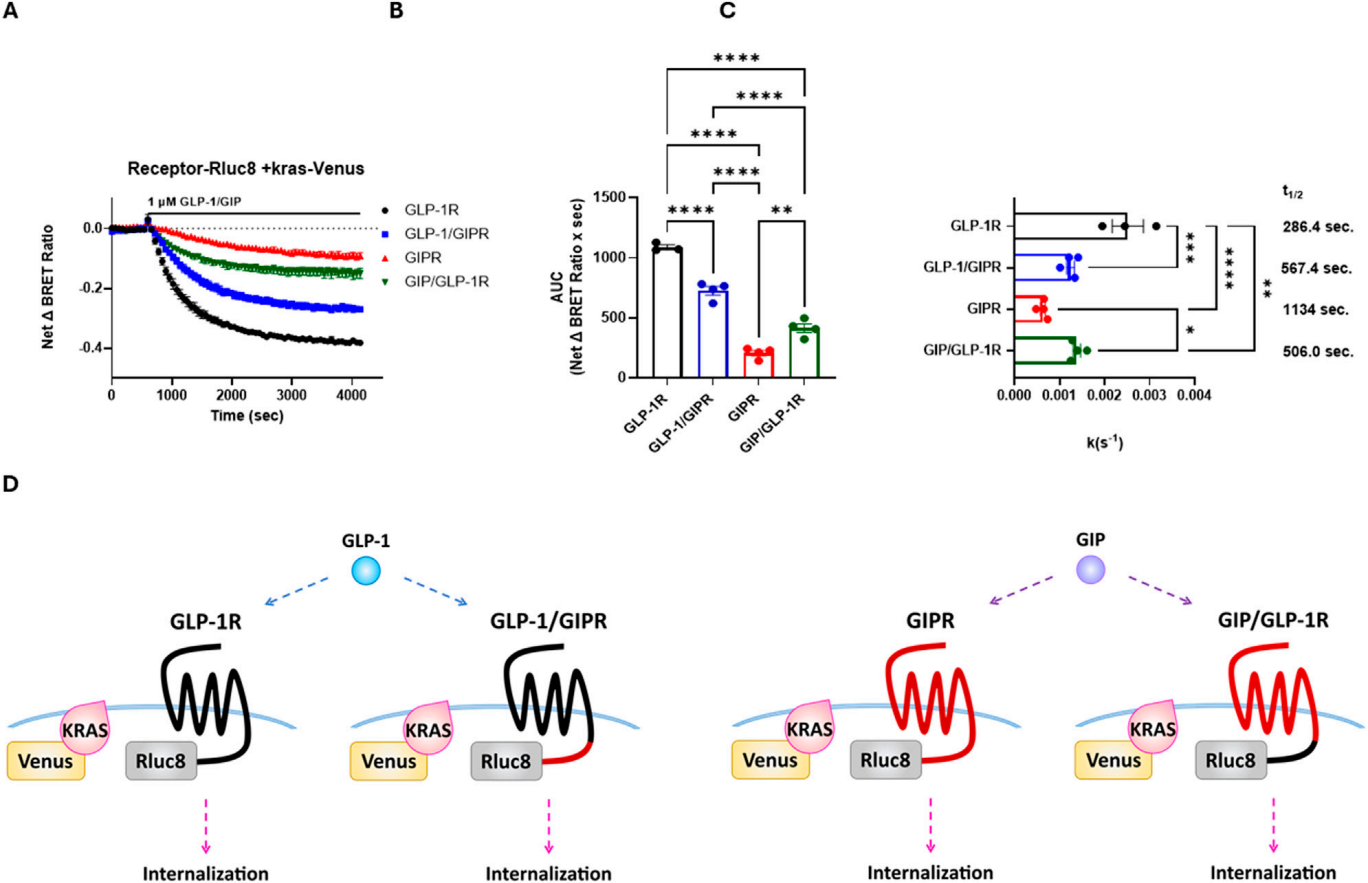
The replacement of the C-terminal region of GIPR with that of GLP-1R increases the rate of agonist-stimulated receptor endocytosis, and the reciprocal substitution decreased the rate of endocytosis. Receptor endocytosis was assessed as a loss of BRET between RLuc8-labeled receptor and Venus-KRAS expressed in HEK-293 cells. GLP-1R and GLP-1/GIPR were stimulated with 1 μM GLP-1, whereas GIPR and GIP/GLP-1R were stimulated with 1 μM GIP. **(A)** Loss of the BRET signal over time between Venus-KRAS and RLuc8-labeled wild-type and chimeric receptors. **(B)** Extent of receptor endocytosis expressed as an area under the curve. The replacement of GLP-1R’s C-terminal tail with that of GIPR significantly (*P* < 0.0001) inhibited receptor endocytosis. In contrast, the reciprocal substitution significantly (*P* < 0.01) enhanced receptor endocytosis. **(C)** Rate of receptor endocytosis. GIP-stimulated internalization of GIPR occurred at a significantly (*P* < 0.01) slower rate than GLP-1-stimulated internalization of GLP-1R. The replacement of the C-terminal tail of GIPR with that of GLP-1R significantly (*P* < 0.05) increased the rate of internalization, but not to the rate of wild-type GLP-1R. Conversely, the replacement of GLP-1R’s C-terminal tail with that of GIPR significantly (*P* < 0.001) decreased the rate of internalization. **(D)** Schematic representation of the chimeric receptors, BRET pairs, and ligands used in **(A–C)**. Data represent the mean ± S.E.M displayed as error bars, from at least three independent experiments, each performed in triplicate.

Agonist-stimulated receptor internalization was assessed as a loss of BRET between the RLuc8-labeled receptor and membrane-targeted Venus-KRAS. GLP-1 treatment resulted in a robust, dose-dependent, and rapid loss of GLP-1R surface expression, whereas treatment with GIP resulted in a significantly (*P* < 0.001) smaller loss of GIPR surface expression than that with GLP-1R, which also occurred at a significantly (*P* < 0.001) slower rate. The replacement of the C-terminal region of GLP-1R with that of GIPR resulted in the significant impairment of agonist-induced receptor endocytosis in terms of the extent (*P* < 0.001) and rate (*P* < 0.005), as well as potency (*P* < 0.05), but not to the same degree as that of wild-type GIPR. Conversely, the reciprocal substitution in GIPR significantly enhanced both the extent (*P* < 0.005) and rate (*P* < 0.05) of agonist-stimulated receptor internalization (Figures 5A–C; Table 3; Supplementary Figure 1).

**TABLE 3:** Agonist-induced internalization of RLuc8-labeled receptors.

		Venus-KRAS	Venus-KRAS	Venus-KRAS	Venus-KRAS
Receptor	*Agonist*	*AUC*	*Rate k(s* ^*-1*^ *)*	*Fold-change from baseline*	pIC_50_
GLP-1R	*GLP-1*	1,088 ± 19.9 _(3)_	0.003 ± 0.0003 _(3)_	−0.23 ± 0.01 _(4)_	7.95 ± 0.07 _(4)_
GLP-1/GIPR	*GLP-1*	726.8 ± 36.0 _(4)_ ^a^ ^,^ ^e^	0.001 ± 8.7e^−005^_(4)_ ^b^	−0.16 ± 0.02 _(3)_ ^d^ ^,^ ^f^	7.54 ± 0.04 _(4)_ ^d^
GIPR	*GIP*	207.3 ± 22.5 _(4)_ ^a^	0.0006 ± 5.3e^−005^_(4)_ ^a^	−0.07 ± 0.02 _(4)_ ^a^	7.32 ± 0.112 _(4)_ ^a^
GIP/GLP-1R	*GIP*	415.2 ± 36.3 _(4)_ ^a^ ^,^ ^g^ ^,^ ^i^	0.001 ± 8.1e^−005^_(4)_ ^c^ ^,^ ^e^	−0.11 ± 0.2 _(3)_ ^e^	7.56 ± 0.04 _(4)_ ^d^

The mean ± SEM is shown from at least three independent experiments (the number of experiments is shown in parentheses) performed in triplicates. The area under the curve (AUC) is expressed as the net change in BRET ratio × seconds. *p*IC_50_ refers to −log IC_50_/M. The *E*_*MAX*_ value indicates the maximum BRET signal expressed as fold-change from baseline.

^a^ *P* < 0.001.

^b^ *P* < 0.005.

^c^ *P* < 0.01.

^d^ *P* < 0.05 significantly different from wild-type GLP-1R.

^e^ *P* < 0.001.

^f^ *P* < 0.005.

^g^ *P* < 0.01.

^h^ *P* < 0.05 significantly different from wild-type GIPR.

^i^ *P* < 0.001 significantly different from chimeric GLP-1/GIPR.

## 4 Discussion

In this study, we investigated the role of the C-terminal regions of GLP-1R and GIPR in cell signaling and endocytosis using chimeric receptors, where the C-terminal region of one receptor was substituted with that of the other. A chimeric receptor approach has been used previously by other groups to define the different regions of the glucagon family of GPCRs responsible for their selective binding of, and activation by, their endogenous ligands (Xiao et al., 2000; Runge et al., 2003). This two-step binding process, where the C-terminal region of the peptide ligand binds to the N-terminal domain of the receptor, allowing the N-terminal region of the peptide ligand to interact with the helical region of the receptor and resulting in receptor activation, has since been confirmed by numerous structures determined by X-ray crystallography and cryo-EM (Underwood et al., 2010; Jazayeri et al., 2017; Johnson et al., 2021). In contrast, much less is known about the molecular determinants that regulate G protein recruitment, receptor desensitization, internalization, and G protein-independent signaling within this family of receptors.

BRET-based mini-G protein recruitment assays were used to investigate the impact of swapping the C-terminal regions of GLP-1R and GIPR on G protein-dependent signaling. Both GLP-1 and GIP recruited Venus-mG_αs_ to their NLuc-labeled receptors in a dose-dependent manner. Although GIP was more potent in this regard, it was not significantly so. When measured as a fold-change over non-stimulated, GIP was significantly less effective than GLP-1 at recruiting Venus-mG_αs_ to its receptor. A similar pattern was observed when Venus-mG_αq_ recruitment was investigated, although in this case, no significant difference was found in E_*MAX*_ (Table 1). GIPR’s reduced ability to recruit both G_αs_ and G_αq_ compared to that of GLP-1R has been reported in previous studies, both in HEK-293 T cells and INS-1 832/3 cells (Jones et al., 2021; Manchanda et al., 2023). These results are initially surprising as GIP contributes more than GLP-1 to the incretin effect in healthy individuals (Gasbjerg et al., 2020). Manchanda et al. (2023) observed no difference between GIP- and GLP-1-stimulated cAMP production or insulin release from INS-1 832/3 cells despite reduced G_αs_ recruitment to GIPR. A possible explanation for these conflicting results is that GIPR is subject to less desensitization than GLP-1R.

Substituting the C-terminal tail of GLP-1R with that of GIPR resulted in a small but significant increase in the extent (*E*_*MAX*_), but not potency, to which GLP-1 stimulated Venus-mG_αs_ recruitment to this receptor (Figure 2A; Table 1). Thompson and Kanamarlapudi (2015a) identified residues 419–430 of GLP-1R’s C-terminus as being required for coupling to G_αs_. Although this region is not completely conserved in GIPR, at least two residues (R411 and L412) are conserved. Nonetheless, based on this study, the GLP-1/GIPR chimeric receptor would be expected to be a poorer recruiter of Venus-mG_αs_ than the wild-type GLP-1R. A possible explanation for the slight increase in *E*_*MAX*_ observed with GLP-1/GIPR is that this chimeric receptor undergoes agonist-induced endocytosis at a slower rate (discussed below). The reciprocal substitution had no significant effect on Venus-mG_αs_ recruitment to GIPR, and neither substitution affected the ability of GLP-1 or GIP to recruit Venus-mG_αq_ to either the wild-type or the chimeric receptor (Figures 2C,E,H; Table 1). An important caveat is that we used mini-G proteins in our assays, which do not consider the role of the βγ-subunits.

For many GPCRs, homologous desensitization first requires the phosphorylation of the active state of the receptor by the G protein-coupled receptor kinase (GRK) family of kinases. This is then followed by the binding of arrestin, which sterically hinders coupling to G proteins, effectively desensitizing the receptor (Krasel et al., 2005). Arrestin can also act as a scaffolding protein, linking the receptor to the cellular endocytic machinery and facilitating internalization (Ferguson, 2001). It is now appreciated that arrestin can also act as a signaling molecule in its own right, linking the receptor to G protein-independent signaling pathways such as mitogen-activated protein kinase pathways (Luttrell and Getsy-Palmer, 2010). Studies performed in various cell types have shown that the activation of GLP-1R by GLP-1 results in the rapid recruitment of arrestin (Jorgensen et al., 2005; Manchanda et al., 2023). Sonoda et al. (2008) demonstrated that the knockdown of arrestin-2 in INS-1 cells impairs GLP-1-mediated insulin secretion, suggesting that arrestin is an integral component of GLP-1R’s signaling pathway. In contrast, arrestin recruitment to GIPR and its potential consequences are less well understood. Although some studies report that GIPR can recruit arrestin, others show that GIPR is either a very poor recruiter or unable to recruit arrestin to any detectable level at all (Al-Sabah et al., 2014b; Ismail et al., 2015; Gabe et al., 2018). The reasons for this disparity are unknown but may be related to the cell context and assay set-up (Al-Sabah et al., 2020). In this study, we could demonstrate a robust, dose-dependent recruitment of NLuc-labeled arrestin to GLP-1R but not to GIPR. As the intracellular C-terminal tail plays an essential role in arrestin binding for many GPCRs and because this region differs between GLP-1R and GIPR in terms of length and potential phosphorylation sites, we hypothesized that substituting the C-terminal tail of GIPR with that of GLP-1R would result in a receptor capable of recruiting arrestin (Tobin et al., 2008). We initially observed no change in arrestin recruitment to GLP-1R with C-terminal substitution (Figure 3A). In this case, the BRET pair was an SYFP2-labeled receptor and an NLuc-labeled arrestin. However, when a different BRET pair was used (RLuc-8-labeled receptor and SYFP2-labeled arrestin), a small but significant decrease in the extent of arrestin recruitment to the chimeric receptor was observed (Figure 3C). This discrepancy between BRET assays could possibly be due to the different sizes of luciferases used in each case (NLuc being 19 kDa and RLuc8 being 36 kDa) (England et al., 2016). Nonetheless, the reciprocal substitution had no significant effect on GIPR’s ability to recruit arrestin, regardless of which BRET-pair was used. This would suggest that while substituting GLP-1R’s C-terminal tail with that of GIPR could moderately impair arrestin recruitment to the chimeric receptor, regions other than the C-terminal tail are more likely to be responsible for the difference in the ability of GLP-1R and GIPR to recruit arrestin observed (Figures 3A, C, E; Table 2).

Following agonist stimulation, most GPCRs undergo endocytosis (Ferguson, 2001). This process serves to regulate the number of receptors expressed at the cell surface and attenuate the cellular response following repeated exposure to an agonist. However, some GPCRs, including GLP-1R and GIPR, continue to signal once internalized (Kuna et al., 2013; Ismail et al., 2016). Most GPCRs undergo endocytosis *via* either a clathrin- or caveolae-dependent pathway (Ferguson, 2001). GLP-1R undergoes rapid internalization following agonist stimulation, which occurs *via* clathrin-coated pits (Widmann et al., 1995). Clathrin-mediated endocytosis (CME) usually requires arrestin to act as a scaffolding protein to link the receptor to the β2-adaptin subunit of adapter protein 2 (AP2), which then targets the receptor to clathrin-coated pits (Kaksonen and Roux, 2018). Interestingly, agonist-induced endocytosis *via* clathrin-coated pits is an arrestin-independent process for GLP-1R (Syme et al., 2006; Thompson and Kanamarlapudi, 2015b). It is possible that both GLP-1R and GIPR can bind directly to the AP2 complex based on the existence of a potential AP2 binding site (YxxΦ) at the interface of transmembrane helix 7 and the C-terminal tail (Kelly et al., 2008). GLP-1R also contains a caveolin-binding motif in intracellular loop 2 and internalizes in a caveolin-dependent manner (Puddu and Maggi, 2021). Based on these conflicting studies, it is possible that GLP-1R can undergo endocytosis through more than one pathway.

The published data regarding agonist-induced internalization of GIPR are just as conflicting as, if not more so than, that for GLP-1R. Some studies demonstrate an arrestin-independent but AP2- and clathrin-dependent internalization mechanism, while others suggest that arrestin is required (Ismail et al., 2015; Gabe et al., 2018). Roed et al. (2014) showed no net change in cell surface expression for GIPR following agonist stimulation. Although this could be interpreted as indicating no internalization, it is also consistent with studies that show that GIPR internalizes slowly but rapidly recycles to the cell surface in a constitutive manner (Mohammad et al., 2014).

Using a BRET-based assay, our data show that GLP-1R undergoes rapid agonist-stimulated endocytosis, whereas a loss of GIPR cell surface expression occurred at a significantly slower rate. The replacement of the C-terminal tail of GIPR with that of GLP-1R significantly increased the rate of receptor endocytosis, which could suggest that this region of the receptor may be involved in GIPR’s reported constitutive recycling. The reciprocal substitution significantly decreased GLP-1R’s rate of internalization but not to the same extent to that of the wild-type GIPR. Interestingly, the kinetics of wild-type GLP-1R internalization fitted best to a two-phase exponential decay model, while all other receptors tested fitted best to a one-phase exponential decay model (Figures 5A–C; Table 2). Taken together, these observations would support the hypothesis that GLP-1R can internalize by more than one mechanism and that regions other than the C-terminal tail may be involved in this process. Moreover, this hypothesis agrees with results reported during the preparation of this manuscript that demonstrate that GLP-1R undergoes endocytosis independent of arrestin and is mediated by both clathrin- and caveolae-dependent mechanisms (Moo et al., 2025).

Substituting the C-tail region of GLP-1R with that of GIPR had no effect on either total expression or cell surface expression, whereas the reciprocal substitution impaired GIPR’s cell surface expression (Supplementary Figure 1D; Figures 4A, B). GIP/GLP-1R’s poor cell surface expression may be a result of increased constitutive endocytosis; alternatively, it may be due to this receptor being retained in either the endoplasmic reticulum or Golgi due to misfolding. The poorer total and cell surface expressions observed with GIP/GLP-1R would be expected to impair agonist-induced endocytosis; however, we observe the opposite. It is possible that this chimeric receptor’s poorer cell surface expression masks an even greater effect on the rate of internalization.

Our results using C-tail swapped GIP receptors are, in part, in agreement with the findings of a recent study that investigated the role of GIPR’s C-terminal tail in signaling and internalization in different species. Rodent GIPR was shown to be less susceptible to internalization and desensitization, as well as weaker at recruiting arrestin, than the human receptor. When the C-tails were swapped between species, there was no significant impact on G protein-mediated signaling, but arrestin recruitment to the human receptor was impaired when its tail was substituted with that of either the rat or the mouse receptor. In the reciprocal experiments, swapping the C-tail of either the rat or mouse receptor with that of the human GIPR improved arrestin recruitment to the receptor and internalization/desensitization (Gasbjerg et al., 2024). Although we observed minimal effects on G protein recruitment with our chimeric receptors and no improvement in GIPR’s arrestin recruitment with GLP-1R’s C-terminal tail, we observed significant differences in rates and extents of internalization.

GLP-1R agonists have been approved for the treatment of T2DM since 2005, but it was only with the approval of tirzepatide (a dual GLP-1R/GIPR agonist) in 2022 that a drug acting on GIPR was used clinically (Le Roux et al., 2023). To what extent GIPR agonism contributes to tirzepatide’s efficacy is still unclear (Gasbjerg et al., 2023). However, it acts as a G protein-biased agonist at GLP-1R, stimulating less internalization than GLP-1 (Willard et al., 2020). Recently, there has been a greater appreciation for targeting GLP-1R endocytosis as a means to improve efficacy. GLP-1R agonists that elicit less internalization produce less nausea, an adverse effect that often limits dosing (Jones et al., 2018). Based on these findings, a greater understanding of the mechanisms by which GLP-1R and GIPR undergo endocytosis is warranted.

The present study may be extended by measuring the interaction between the wild-type and chimeric receptors and arrestin-2 (β-arrestin-1). It would be interesting to investigate whether the C-terminal tail plays a role in differentially recruiting different isoforms of arrestin. In addition, exploring downstream signaling pathways such as cAMP and ERK would enhance our understanding of the role of these receptors’ C-terminal regions in cell signaling.

## 5 Conclusion

In summary, we demonstrate that the C-terminal tails of GLP-1R and GIPR are not the key determinants for their differing ability to recruit G proteins and arrestin. Substituting GIPR’s C-terminal tail with that of GLP-1R significantly increased both the rate and extent of agonist-mediated endocytosis, suggesting a role for GIPR’s C-terminal region in either its slower internalization or rapid recycling back to the plasma membrane. The reciprocal substitution significantly impaired GLP-1R internalization, but not to the same extent as that of wild-type GIPR. These data support the hypothesis that regions other than GLP-1R’s C-terminal tail may also be involved in endocytosis.

## Data Availability

The raw data supporting the conclusions of this article will be made available by the authors, without undue reservation.
