# Supplementary material for: The C-terminal regions of the GLP-1 and GIP receptors are not the key determinants of their differential arrestin recruitment but modulate the rate of receptor endocytosis
Source: Front Pharmacol. 2025 Mar 25;16:1528295. doi: 10.3389/fphar.2025.1528295 (PMC11975949; doi:10.3389/fphar.2025.1528295)
Supplement: Supplementary file 1 [file DataSheet1.docx]

| **Primer Name** | | **Sequence (5'-3')** |
| --- | --- | --- |
| **GLP-1R N-term.** | **Forward** | ACTCACTATAGGGAGACCCAAGCTTATGGCCGGCGCCCCCGGC |
|  | **Reverse** | TGTTGATGAAGCAGTATAATAGGCCACCATCAGCCCCTGGAAGGAGGTGAAGG |
| **GIPR C-term.** | **Forward** | CATATTATACTGCTTCATCAACAAGGAG |
|  | **Reverse** | GCTCCTCGCCCTTGCTCACTCTAGAGCAGTAACTTTCCAACTC |
| **GIPR N-term.** | **Forward** | ACTCACTATAGGGAGACCCAAGCTTATGACTACCTCTCCGATCC |
|  | **Reverse** | TGACAAAGCAGTAGAGGACGCTGACCAG |
| **GLP-1R C-term.** | **Forward** | CGTCCTCTACTGCTTTGTCAACAATGAGGTCCAGC |
|  | **Reverse** | GCTCCTCGCCCTTGCTCACTCTAGAGCTGCAGGAGGCCTGGCA |

**Supplementary Table 1. Primers used to generate the chimeric receptors GLP-1/GIPR and GIP/GLP‑1R.** GLP‑1R N-term. (Forward and reverse) and GIPR N-term. (Forward and reverse) were used to amplify the N-terminal and transmembrane region of the respective receptor and GLP-1R C‑term and GIPR C‑term were used to amplify the C-terminal tail of the respective receptor.


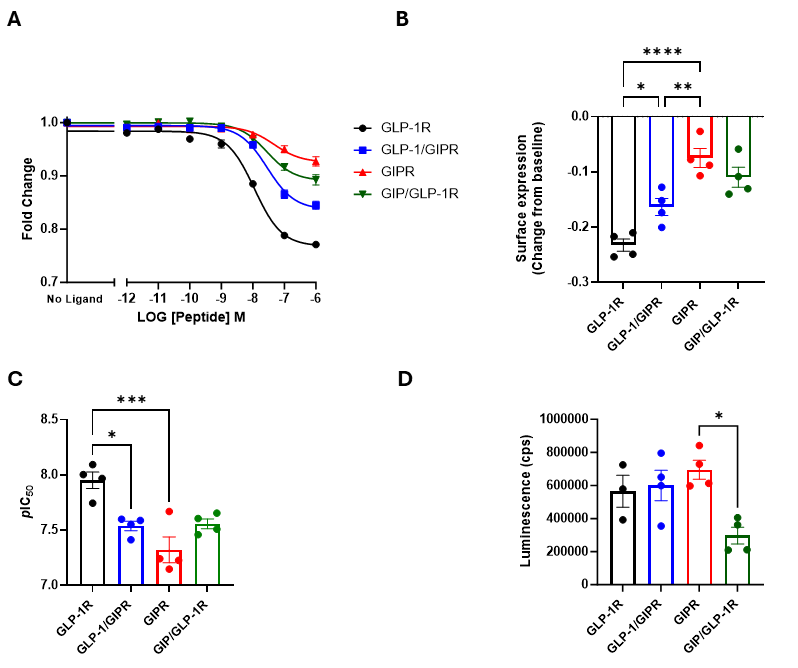


**Supplementary Fig. 1.** **Receptor endocytosis assessed as a loss of BRET between the Rluc8‑labelled receptors and Venus-kras expressed in HEK 293 cells.** GLP-1R and GLP-1/GIPR were stimulated with GLP-1 and GIPR and GIP/GLP-1R were stimulated with GIP. **A.** Concentration‑dependent loss of BRET between Rluc8-labelled receptor and Venus-kras expressed as fold-change from baseline. **B.** The extent of receptor endocytosis expressed as fold-change from baseline. Replacement of the GLP-1R's C-terminal tail with that of GIPR significantly (*P*<0.05) inhibited receptor endocytosis. In contrast, the reciprocal substitution (GIPR and GIP/GLP-1R) did not significantly enhanced receptor endocytosis. **C.** *p*IC_50_ values derived from **A**. where *p*IC_50_ and refers to -log IC_50_/M. **D.** Expression of wild-type and chimeric receptors expressed as total luminescence. Only GIP/GLP-1R was expressed at a significantly (*P*<0.05) lower level than wild-type GIPR, (*P*<0.05). The mean ± SEM displayed as error bars from at least three independent experiments performed in triplicate.
